# Information System as part of epidemic management in Burkina Faso: from plan to reality (Field Findings)

**DOI:** 10.1186/s12889-022-14072-1

**Published:** 2022-09-12

**Authors:** Cheick Omar Diallo, Karin Linda Schiøler, Helle Samuelsen, Koine Maxime Drabo

**Affiliations:** 1 University of Ouagadougou, 09 P.O. Box: 480, Ouagadougou 09, Burkina Faso; 2 Global health section, Department Public Health, University of Copenhagen (UCPH), Copenhagen, Denmark; 3 Department of Anthropology, University of Copenhagen (UCPH), Copenhagen, Denmark; 4 Public health, University of Ouagadougou, Ouagadougou, Burkina Faso

**Keywords:** Information system, management, Burkina Faso

## Abstract

**Background:**

Health information systems (HIS) in most developing countries face many challenges. In view of the recurrent weaknesses in preparedness and response during the management of epidemics, we have examined the organization and functioning of the health information system in Burkina Faso.

**Methods:**

We conducted a cross-sectional study from January 1, 2020 to March 31, 2020 including a review of HIS documents, key informant interviews and direct observations. The study was conducted at the public primary health care (PHC) and community level of Bama and Soumagou, in the rural health districts of Dandé and Tenkodogo. Study participants included community-based health workers (CBHWs) and health workers in the PHC areas, community-based organization animators (CBOAs), CBO monitoring-evaluation officers and members of the District management team (DMT).

**Results:**

While reporting forms used in all health facilities are standardized, they are not necessarily well understood at community level and at the health centers. Reports prepared by CBHWs are often delayed by the head nurse at the primary health care service. Case definitions of epidemic diseases are not always well understood by community-based health workers and front-line health workers.

**Conclusion:**

The health information system in Burkina Faso can be improved using simple strategies. There is a need to hold regular training/refresher sessions for agents involved in surveillance and to ensure the development of simplified case definitions for emerging diseases and/or diseases of public health interest for community use. Furthermore, existing epidemic management committees need to be revitalized.

## Background

The role of health information systems (HISs) is to produce, analyse and disseminate reliable health data in a timely manner [1]. However, few developing countries have a sufficiently robust and effective HIS to fulfil this role. Multiple constraints including lack of adequate health information policies, limited and unequal distribution of available resources, disorganization and fragmentation due to administrative, economic, or donor pressures, as well as the absence of standards are common explanations for fragile HISs [2–4]. Integrated Surveillance and Response (IDSR) refers to a strategy that aims to strengthen the HIS through improved disease surveillance as well as laboratory and response capacities at community, district and national levels [5]. Notably, the strategy was adopted by all member countries of the WHO-Afro region in 1998 [6–8].

Burkina Faso, in West-Africa, is facing challenges such as high mortality rates (11.8 ‰), especially among mothers and children [9]. Notably, 24% of all under-5 deaths (102 per 1000 live births in 2012) are caused by malaria, while 18% of all under-5 deaths are attributed to acute lower respiratory infections [10]. There are high burdens of child morbidity and mortality due to recurrent epidemics of measles and meningitis, while epidemic transmission of diseases such as yellow fever, dengue and, most recently COVID-19, pose additional health threats across all age groups [9–11]. Faced with these health challenges, the national health policy of Burkina Faso aims to improve the health status of the population by paying specific attention to the reduction of mother and child mortality rates, as well as targeting high-mortality conditions namely: malaria, tuberculosis, HIV and malnutrition [12].

The national HIS is part of the strategy to achieve this goal and includes all primary, secondary and tertiary health facilities of the country [13]. Non-governmental organizations (NGOs) also contribute to the HIS through their support to various community health structures. The HIS in Burkina Faso has six components built around an information system for i) routine health service reports, ii) epidemiological surveillance (early warning system), iii) program management, iv) administration and resource management, v) community-based surveillance, and vi) periodic surveys and studies [14]. In addition, the IDSR strategy of Burkina Faso aims to support and strengthen the national HIS for all priority diseases, including those with high epidemic potential. The strategy is designed to assist the systematic processes for collecting, analysing, disseminating, and using health-related data dispatched from the primary health care centers (PHCs) up to the Ministry of Health. Districts are the most decentralised operational entities of the health system. It comprises two levels of care, the first one being the Primary Health care center (PHC) is the closer to the population within its range, and has a minimum package of curative, preventive and promotional activities. The HD also comprises the district hospital, which is the referral level with a complementary package of activities [15].

Within the framework of health information management in general, and for epidemics in particular, the managers at the PHC level are to interact closely with community-based health workers (CBHWs), community-based organization animators (CBOAs) and the people in charge of monitoring and evaluating the CBOAs. Community-based surveillance involves community representatives, neighbourhood leaders, village chiefs, etc. Their role is to provide PHC managers with quality health surveillance information that is useful at all levels of the health system for planning and decision-making concerning important public health events and epidemics [16]. The aim of the community-based system is thus to improve public health surveillance and response to health events in the community by linking communities more directly to their local health facilities [17].

The WHO Health Metrics Network (HMN) is based on the principle that collecting better health information leads to making better decisions, which lead to better health [2]. The HMN Framework describes the six components of a HIS and standards required for each component. Based on these six components, a HIS can be subdivided into three categories: i) inputs, ii) processes and iii) outputs. Inputs refer to resources while processes refer to how indicators and data sources are chosen and how data are collected and managed. Outputs refer to the production, dissemination and use of information [2]. Defining what constitutes a HIS and how its components interact to produce better information for improved health and decision-making allows for a better understanding of the HIS.

In this study, we use the HMN framework to examine the organization, functioning and interaction of the two surveillance components of Burkina Faso’s HIS, namely, the epidemiological surveillance and the community-based surveillance. The aim of this study was to compare the official reporting system (HIS) with the actual reporting practices in the rural Districts of Dandé and Tenkodogo.

## Methods

### Study design and study sites

We conducted a cross-sectional study from January 1, 2020 to March 31, 2020 at the public primary health care (PHC) and community level of Bama and Soumagou in the rural health districts of Dandé and Tenkodogo, respectively. The two health districts are located in “Hauts bassins” (Dande) and the “Center-East” (Tenkodogo) regions of Burkina Faso. Given their relative proximity to Mali (Dande), Togo and Ghana (Tenkodogo) and the continuous cross boarder movement of humans, animals and goods, these districts are considered as areas of increased risk of epidemic disease transmission, which make them relevant study sites for assessing the surveillance components of the HIS.

### Participants and sampling

Using a purposive sample, we included HIS members responsible for health surveillance in general and epidemic surveillance, specifically, including all Community-based health workers (CBHWs) and health workers in the PHC area. Additional personnel included community-based organization animators (CBOA), CBO monitoring-evaluation officers^1^ and members of the District management team (DMT) directly involved in health surveillance. We interviewed a total of forty-one actors (Table 1).

**Table 1:** Summary of secondary data sources in Dandé and Tenkodogo health Districts, Burkina Faso

Official documents	
Standards	Operational
Technical Guide for Integrated Disease Surveillance and Response in Burkina Faso. Section 1 to 8: Steps in surveillance.	TLOH (weekly reports)
	Notification form
Technical Guide for Integrated Disease Surveillance and Response in Burkina Faso. Section 9: Guidelines for priority diseases, illness and other public health events	Activities reports (CBHWs monthly reports, PHCs monthly reports, annual reporting…)
Job description of agents in charge of the HIS.	Monitoring reports
Training module for community-based health workers.	2018 statistical yearbook
Guideline for completion of the monthly activity report of the community-based health worker.	2019 statistical yearbook
Guideline for completion of the monthly activity report of Primary health care centers/dispensary/maternity ward/birth clinic.	Dandé health District 2018 action plan
	Tenkodogo health District 2018 action plan
Guide to filling in data collection tools- PHC, CM/CMA and District executive team level.	Dandé health District 2019 action plan
National guide for CBHWs supervision.	Tenkodogo health District 2019 action plan

*CM/CMA* Medical center, *PHC* Primary health care center, TLOH: Weekly report

### Study items

**Fig. 1:**
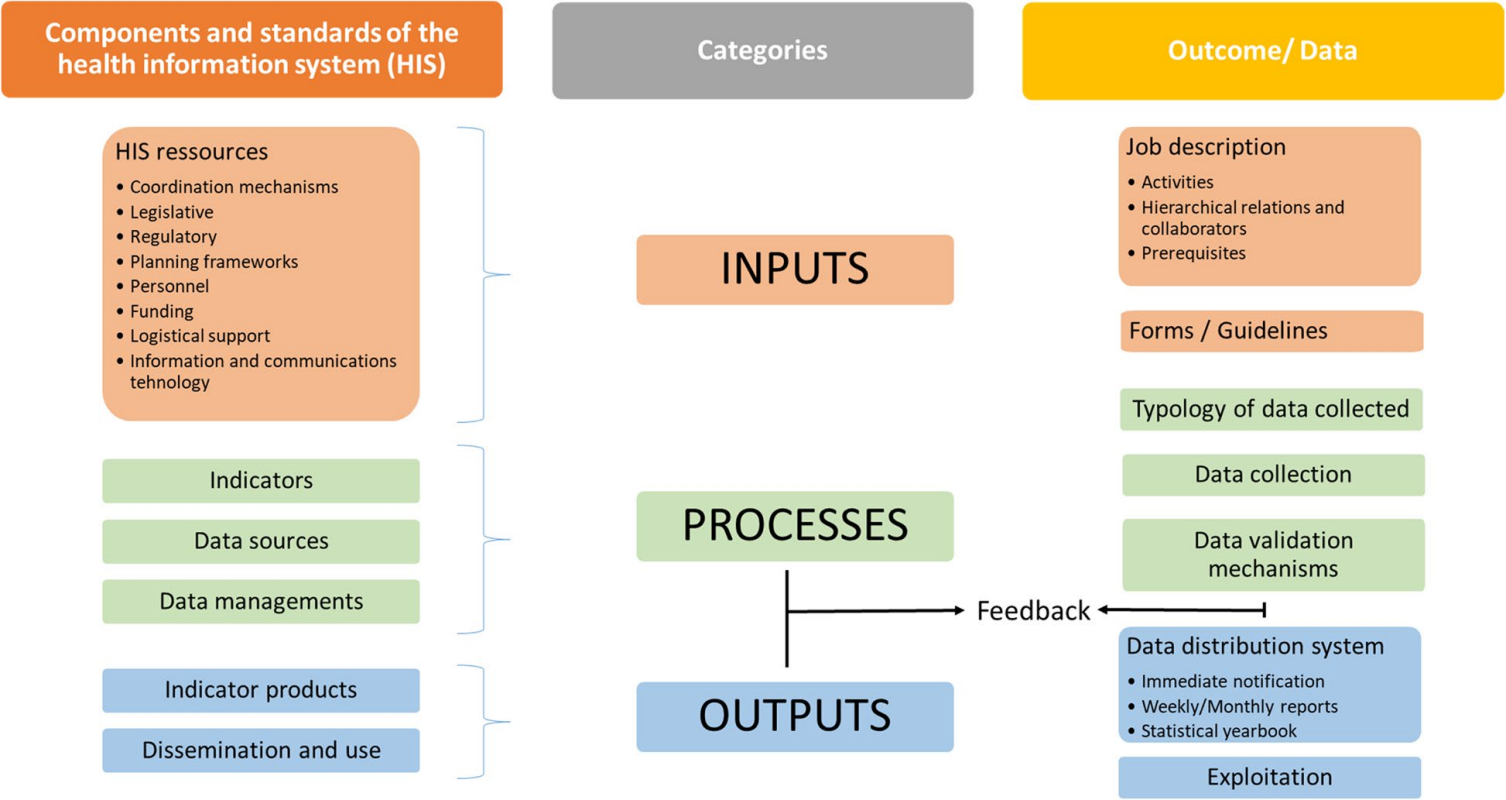
Categories examined in the study. (Adapted from WHO framework and standard for country health information systems)

The goal of the WHO HMN is “to increase the availability, accessibility, quality, and use of critical health information for decision making at the national and global levels” [2]. Referring to the HMN framework (2012), we assessed the following:

i) Inputs, including the legislative, regulatory and planning frameworks required to ensure the full functioning of the health care system, as well as the resources required to ensure that such a system is functional. These resources include personnel, funding, logistical support, information and communications technology (ICT), as well as coordination mechanisms. Specifically, we looked at the activities by people involved in surveillance, their hierarchical relations and collaborators, as well as their prerequisites to identify the items in the input.
ii) The process, including indicators, data sources and data management, involving all aspects of collection, processing, storage and quality assurance, analysis and communication. More precisely, we focused on the types of data collected, data production and data validation mechanisms.
iii) Outputs, including production, dissemination and use of information. In the study, we focused on the data distribution system and use (Fig. 1).

### Data collection

We collected data using the following methods: i) Key informant interview: Primary data were collected through key informant interviews based on semi-structured questionnaires and ii) Observation: through direct observations of the facility setting using a thematic checklist. iii) Document review: Secondary data were collected from official surveillance documents including job description of agents in charge of the HIS in Burkina Faso, training module documents for CBHWs, guidelines for completion of the monthly activity report of the CBHWs, and activity reports of the PHC. Data were also collected from documents produced in the field as part of data management including monthly activity reports by CBHWs, activity reports of the PHC and the statistics of health facilities.

### Data analysis

All primary and secondary data were thematically analysed for comparison between the planned framework and the actual activities taking place at the different levels of the HIS. We identified specific analytical categories: surveillance activities, hierarchical relations and community-level collaborations, prerequisites, types of data collected and data production, data validation mechanisms, data distribution system and exploitation. We used distinctive (technique by technique) or convergent analysis when appropriate.

## Results

### Burkina Faso Health information system flow diagram

**Fig. 2:**
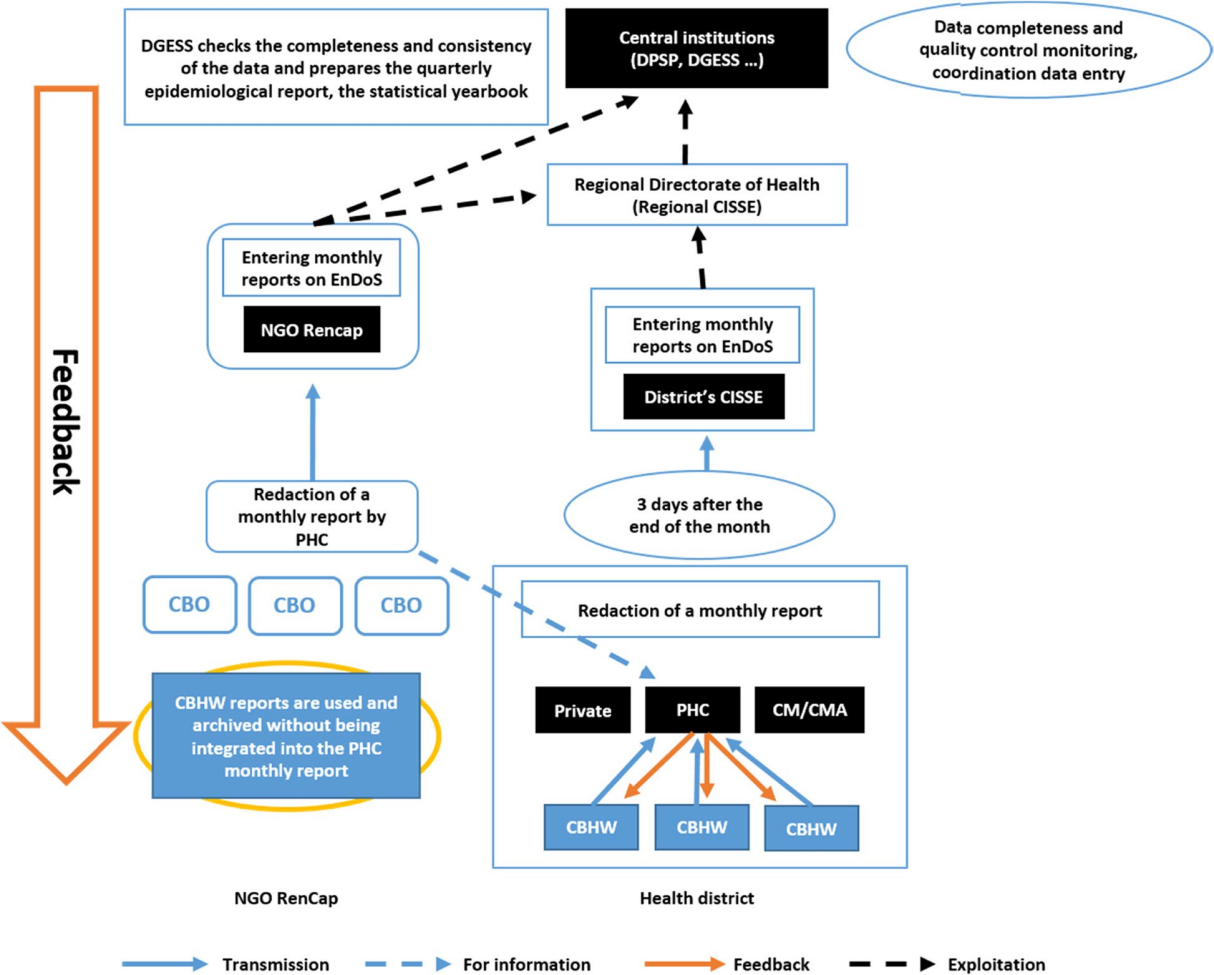
Burkina Faso Health information system flow diagram in health districts. (Source: Adapted from Ministry of health). CM/CMA: Medical center. CISSE: Health information and epidemiological surveillance center/officer. CBO: Community Based Organization. CBHW: Community Based Health Worker. DGESS: General directorate of studies and sectorial statistics. DPSP: Directorate for the Protection of Population Health. EnDoS: Health data warehouse. NGO: Non-governmental organization. PHC: Primary health care center

Figure 2 provides an overview of the health system flow of information in health districts in Burkina Faso.

### HIS inputs

#### Surveillance activities

The different surveillance activities at each level of the health system were clearly defined in the technical guide for Integrated Disease Surveillance and Response and by the job descriptions for the different actors engaged in the HIS [7, 16, 18–23] (Table 2). Notably, at community level, CBHWs were expected to collect information related to the management of resources such as drugs, rapid diagnostic tests, bed nets, etc., curative and preventive activities such as the number of consultations, debates, counselling sessions, home visits, and notification of births/deaths in households or rumours thereof. The CBHW reports suspected cases of notifiable diseases, actively tracks patients lost to follow-up treatment and participates in monthly meetings with the head nurse (ICP) at the local health center. CBO monitoring and evaluation officers were instructed to analyse, interpret and archive all data reported by the CBOAs and to submit CBO progress reports to the Health District. They were also required to provide feedback to CBOs. CBOAs were responsible for outreach activities regarding disease control, sexual and reproductive health, and child nutrition.

**Table 2:** Overview of study participants in Dandé and Tenkodogo health Districts, Burkina Faso

Study Participants	
Profiles	Number included/total number
**District Management Team (DMT)**	
District medical officer (MCD)	2/2
District’s health information and epidemiological surveillance officer (CISSE)	2/2
District hospital manager	1/2
Laboratory manager	2/2
**Primary health care center staff (PHC)**	
Head nurse (ICP)	2/2
ICP colleagues (other health workers)	8/8
***Community “rapporteurs“***	
Community based health worker (CBHW)	21/22
Community-based organization (CBO) animator	1/-
Community-based organization (CBO) monitoring and evaluation officers	2/-
**Total**	41

At the primary healthcare level, the Head Nurse (ICP) held numerous responsibilities including the investigation of unusual events based on rumours and unexplained deaths, as well as the immediate notification of suspected cases of disease. The ICP was also in charge of the supervision of CBHWs as well as the provision of materials, guidelines and collection forms to the CBHWs as needed. Moreover, the ICP was responsible for validation, analyses, interpretation and use of health information data for local decision-making and for compilation and submission of weekly (TLOH) and monthly surveillance reports to the District level. ICPs were to participate in epidemiological surveys organized in the District, to establish the epidemiological profile of the health center and provide feedback to the community (through the CBHWs), the health facility management committee (CoGes), the municipality and religious leaders.

In accordance with available guides and job descriptions, the key informant interviews revealed that surveillance actors are trained at the time of recruitment and that surveillance activities are carried out according to plan. However, identification of notifiable diseases are hampered, as some CBHWs or ICPs do not fully understand the case definitions written in: *“…there are items we do not understand well […] or it is a lack of training. There are items that are confusing because if we argue about an item, it is not clear” (*DMT member, male, 7 years work experience). Inspection of the monthly reports submitted by CBHWS also revealed a main focus on reproductive health, malaria and nutrition and that not all CBHWs are able to fill out the surveillance report forms themselves; they request help from the ICP.

#### Hierarchical relations and community-level collaborations

The lines of authority and decision-making power was clearly described in the analysed document (job description of agents in charge of the HIS), as were the collaborative arrangements within and between the different levels of the HIS. At community level, with a mean of 10 per PHC in Tenkodogo and 4 per PHC in Dandé (according to 2019 statistical yearbook), CBHWs remain under the authority of the ICP, while collaborating with other CBHWs, the CBOA and the CBO monitoring and evaluation officer. Moreover, CBHWs collaborate directly with community leaders, traditional healers, the head of the village development council, district delegates, delegates of villages in the health area, and the head of the PHC management committee (CoGes). The CBOA is under the responsibility of the CBO monitoring and evaluation officer (incl. annual reporting of activities). He/she collaborates with village CBHWs in the health area (awareness raising activities). The CBO monitoring and evaluation officer collaborates with CBHWs, PHC health workers, the health information and epidemiological surveillance center/officer (CISSE) at District and Region levels.

At the PHC, the head nurse (ICP) reports to the District medical officer, the District CISSE and the administrative and communal authorities. The ICP collaborates with other health workers at the PHC, community actors of the HIS as well as traditional healers in the PHC area..

Our key informants confirmed the hierarchical and collaborative relationships as described above. However, some community “rapporteurs” (CBHWs) and the DMT noted that potential collaborators such as traditional healers or primary school teachers are not sufficiently involved in disease surveillance despite their strong influence - as compared to CBHWs - on social mobilization and case referral.

#### Prerequisites: educational/literacy level at recruitment, work resources and incentive

The documents analysed show that the minimum educational/literacy requirement for recruitment of CBHWs, CBOAs and CBO monitoring and evaluation officers is the primary school certificate. The ICP must hold the basic nurse or midwife training degree. The CBHW work materials include checklists, consultation registers, reference and counter-reference sheets, community resources (drugs, rapid diagnostic tests, bed nets etc.), cell phones for communication with health workers (for case identification), a megaphone and a bicycle At community level, the types of incentive planned for health workers are mainly paid in-service training, regular supervision, study trips, congratulation letters , honorary distinctions. CBHWs receive monthly financial incentive from the state and from the Global Fund to Fight AIDS, Malaria and Tuberculosis.

The ICP has a mobile phone connected to the District fleet, allowing him/her to transmit the PHC’s TLOH data. He/She may go on study trips, receive in-service training, letters of congratulation from supervisors (District medical officer and / or the municipal authority), honorary distinctions and promotion to District level.

According to some CBHW interviews, the educational/literacy criteria are not applied during the recruitment of CBHWs. This explains the difficulties observed for CBHWs when it came to reading and/or understanding HIS documents: low access to the content of the documents and the reasoning model/logic. CHBWs also experience difficulties in receiving their regular financial incentives. “*People are not motivated [CBHWs]; it has been shouted all over the place they are paid 20,000 CFA per month, but actually I don't know if in 2019 they got anything*” (DMT member, male, 16 years’ work experience). The non-payment of allowances often leads to difficulties in obtaining telephone credit while CBHWs are not included in the District’s phone fleet. CBHWs may also lack petrol for traveling to some distant areas places after receiving notification about suspected cases . Yet the phone network coverage is often poor or non-existent.

### HIS process

#### Types of data collected and data production

According to the procedures, the CBHW must use standard forms and produce a CBHW report to the local ICP. The ICP must produce a weekly TLOH report and a monthly PHC activity report to the CISSE. In the case of a suspected notifiable disease, the ICP is required to notify the CISSE immediately by phone and complete a follow-up notification form. Verification of collected data (TLOHs, monthly activity reports and notification forms) is the responsibility of the ICP, while the District CISSE aggregates all reports and forms. The CBHW report is submitted after the 25th of the month and no later than the last day of the month covered by the report. TLOHs are sent every Monday at 10 a.m. at the latest and notification forms must be submitted within 24 hours for each suspected case [21].

According to some study participants at the PHC and DMT, actors at community level or even by newly assigned staff at the PHC level do not always understand the standardized report . One reason evoked by a DMT-member (male, 7 years’ work experience) is the lack of training or retraining on surveillance, its importance and its procedures: *“We just need to train people. [The ministry of health] have to train people in analysis and reporting techniques and then harmonize the items. Because if the items are harmonized, if the definitions are harmonized and people are trained, we have the chance to have a single information. This must not cause confusion. That means that when we say a definition, it must be as simple and as clear as possible, so that people do not get confused. And the data collection form must be really clear*”.

It was noted that CBHW reports are often compiled and transmitted to District level with a 2 to 5 days’ delay , sometimes without any check of data completeness by the ICP. The tedious verification of data collected in the monthly PHC activity report (consisting of 38 sheets) including verification of missing case information, such as patient data concerning age, socio-professional and vaccine status, often overloads the ICP. This workload impedes the supervision of clinical activities, of the bi-monthly CBHW supervision, and the investigation of disease rumours. At District level, the CISSE aggregate all data from the TLOH and PHC reports in an Excel sheet, while notification forms of suspected cases are recorded in different electronic databases (IDSR^2^, e-Surveillance or STELaB^3^) for follow-up.

The _ICP usually refers to directives coming from the central level, however there is no formal procedure for verifying the transmitted data. According to a District medical officer (DMO), the local databases set up by the District CISSE do not always undergo corrective checks after the final official validation.

#### Data validation mechanisms

Formally, at District level, the CISSE and the DMO, validate the disease indicators provided in the CBHW’s report, the ICP’s weekly reports and notification forms. They also verify the absence of outliers, completeness and promptness of data, every week and quarterly. A final validation is made every six months with the regional health team and central level [21].

The main difficulty observed in the field was the lack of formal procedures on how to verify and correct the reported data, and the failure to maintain regular epidemic management meetings. As exemplified by a DMT member (male, 10 years work experience): *“We no longer hold meetings because they are simply meetings that are no longer funded... There are no resources to hold provincial epidemic committee meetings. Moreover, when you take for example the District Health Council (CSD), it is the highest statutory - body in the district, which is chaired by the “haut-commissaire” [High Commissioner]. Therefore, people are trying to juggle. If they have the funding for a meeting for example on nutrition...they seize the opportunity to hold their CSD*”. Which means some meetings, do not always involve all the actors concerned.

### Finding related to output

#### Data distribution system and exploitation

According to available HIS documents, the PHCs transmit the surveillance data to the District CISSE by telephone and in paper format. From there, the data are disseminated at regional level, and sent to the managers at central level (Directorate for the Protection of Population’s Health - DPSP and the Directorate of Sectorial Statistical Studies - DGESS) by telephone and e-mail. Feedback is provided by the central level through quarterly epidemiological reports, statistical yearbook, phone calls, electronic surveillance (laboratory results) and e-mails.

Based on our observations and key informants’ interviews, the distribution flow of data mentioned above is well implemented across all HIS levels. However, whereas the Health districts have a mobile phone fleet covering the PHCs, not all CBHWs have access to the fleet, so they have to use their own resources (cell phones and telephone credits) to communicate with the PHC, which hampers their reporting efforts. At District level, the sharing of data from the CBHW reports through the Health data warehouse (EnDoS) is incomplete, as the 2018 statistical yearbook shows that only 16.0% and 64.7% of the CBHW reports were available from the health districts of Tenkodogo and Dandé, respectively [15].

In addition, we observed important discrepancies between the weekly data reported by ICPs and the number of suspected measles (9.2%) and meningitis (33.8%) cases recorded in the IDSR database in 2018. According to a DMT member, this is due to poor archiving and a lack of corrective actions of local databases after the final data validation sessions. In some cases this may also be due to the fact that certain staff members lack the skills in to use computerized platforms managing descriptive lists, which leads to duplicates or false cases. Among the reported cases registered in the disease surveillance databases (IDSR), we found that missing data most often relate to socio-professional classification and patient history.

The District CISSE provides laboratory feedback to the ICP directly by phone, but only in the case of positive test results. All negative test results are sent to the PHC’s letterbox located within the District itself. In some cases this may be as far as 50 km from the PHC. In addition, as an ICP pointed out, due to medical confidentiality, feedback on notified cases provided by the PHC to CBHWs is limited to collective sensitization activities.

## Discussion

This study aimed to examine the organization and functioning of the HIS in Burkina Faso in terms of expected and actual activities by comparing official documents to primary information obtained from key informants and direct observations. Based on the WHO HMN frameworks, we focused specifically on inputs, processes and outputs.

### Limitations of the study

The study has some limitations, including potential bias due to the purposive selection of the two Districts and participants, as well as the risk of not being representative of health districts in Burkina Faso. Moreover, private health facilities, religious-owned health services and actors, and animal health services were not included in this study. However the number of such services are limited and they are mainly concentrated in urban areas. They could have provided additional information and given a more complete view of epidemic surveillance as part of the HIS. Nevertheless, the results obtained offer inputs for reflection on improvements strategies in Burkina Faso and similar settings.

### Main gaps between the planned and existing system

#### Input category

Regarding the activities relating to community-level surveillance in Burkina Faso, the official forms for reporting epidemic diseases and events in the community are in French, which poses a challenge for some CBHWs in terms of reading and completion. Our study also revealed, that some forms contain certain biomedical terms that do not align with local symptoms and disease perceptions. This may result in CBHWs’ failure to detect and report disease cases as required. In their evaluation of the surveillance systems in Burkina Faso, Geers and colleagues (2018) reported that some CBHWs suggested the use of community definitions of meningitis and measles for better comprehension [24]. Moreover, in community settings, the registration of deaths by the civil registry office in municipal councils is often insufficient and verbal autopsies on causes of death are not carried out systematically [25]. This may partly be explained by the lack of collaboration between the Ministry of health and the Ministry in charge of civil registration. That may also question the reliability of the detection process and hamper the functioning of early warning systems to contain epidemic diseases.

We noted that most of the CBHWs are primarily trained to provide services to the community for reproductive health, malaria, vaccines and nutrition. Furthermore, the community-based surveillance of epidemic diseases is not well developed in terms of training on case identification and reporting [24]. With the appearance of new epidemic diseases such as dengue and COVID-19, a stronger focus on emerging diseases and CBHWs participation is needed, including improved data sharing, and feedback between the different HIS levels and the community.

At community level, it may be an advantage for example to involve traditional healers and school teachers in a direct collaboration with the CBHWs for epidemiological surveillance. As a matter of fact, the limited engagement of traditional healers and school teachers was documented in the stakeholder analysis of Dandé’s 2018 and 2019 Health District action plan [26, 27]. In a similar setting in Niger, Ndiaye and colleagues (2000) reported that due to cultural beliefs, patients would consult traditional healers before going to health centers, which causes delays in early detection, reporting and investigation of disease cases [28]. According to the World Bank (2012), “Information on the types and volumes of services offered by private facilities is almost non-existent in Burkina Faso. As a result, the role played by the private health care sector is difficult to assess, and the quality of services is difficult to appreciate as well” [29]. As some people prefer to attend private facilities for various reasons, such as faster treatment, better comfort and the availability of specific services, the insufficient involvement of private actors in the surveillance of epidemics may constitute a source of vulnerability for the existing health systems in terms of early detection and notification of epidemics.

The clustering of the actors involved in disease surveillance, as noted in this study, is common in African health systems [30]. Information and communication technologies (ICTs) could improve health system management including the relations between actors [31, 32]. This solution may already be in a process toward implementation with the new WHO e-IDSR strategy [33]. In some countries, an SMS-based mobile phone network has been set up to enable CBHWs to exchange short text messages [34]. Using mobile applications could allow CBHWs to feel part of the system and change their expectations and roles in positive ways and increase their personal commitment [35].

The Ministry of health and the health officials in Dandé and Tenkodogo should strive to improve CBHWs’ understanding of forms and produce simplified case definitions (with less technical terms) for all diseases with an epidemic potential. Therefore, information needs to be formulated according to CBHWS’ level of instruction, and that must be done under the supervision of a district, regional, or central staff [35]. As noted by the USAID, interconnecting forms and electronic platforms of various programs could allow for real-time notification, collection, analysis and use of data on diseases or events for effective public health interventions. Indeed, by training the CISSE and other data managers, such as laboratory technicians, to enter data correctly across existing programs, could improve data quality, as observed in this study. In Tanzania Nsaghurwe and colleagues (2021), showed that it is possible to integrate and share digital data between levels and programs of the health system even when people use different digital tools. Indeed, data entry errors like, for example, a discharge prior to admission dates, were resolved through interoperability filters of the health information exchange system, which spotted such errors and requested corrective actions from the point of data entry [36].

Ideally, information from the community and PHC levels are used for planning and managing of the surveillance system as well as for advocacy and policy development. According to official documents, the head nurse should be able to analyse, interpret and use health information data for local decision-making [16]. In practice, the nurse, and even the DMT, solely refer to directives from the central level in Ouagadougou. They do not have decision-making autonomy regarding the management of problems encountered in their area of responsibility, despite the fact that they carry out annual analyses in which their problems are identified. As reported by Odhiambo-Otieno in Kenya (2005), such centralized decision-making may often overrule or ignore local expectations [37].

Certain actors, particularly ICPs, report to several other actors, such as CBHWs, DMOs and the CISSE. This increases the risk of errors, widens the discrepancy between the TLOH data and the IDSR database, causes duplications during the reporting and delays in the flow of information. Ouedraogo and colleagues (2018) highlight the tedious task of entering and reporting health data, with the risks of duplicate entry in different databases [38]. A number of other factors may explain inadequate reporting, including insufficient quality control by supervisors due to lack of procedures or directives, inexperience of new staff with forms and guidelines, and individual or social factors – such as labour disputes between health workers and the government. In 2019, health workers went on strike for a continuous period of eight months, thus causing a noticeable drop in the year’s statistical reports [13, 39].

Because of the different skill levels between some community “rapporteurs” and the ICP, the interface of their collaboration gets complicated because of the other stakeholders’ inability to understand the forms provided by the ICP or the latter’s inability to explain the forms in an easy language. Schweyer and Cabe (2005) addressed this phenomenon by; for them, “procedures or tools are not those that structure the networks, but a more egalitarian approach between health professionals” [40, 41].

Official surveillance documents mention that the head nurse or any other designated person must file the CBHW monthly report. However, because of PHC understaffing, a single nurse may be linked to CBHWs from several villages and therefore be responsible for multiple reports, which causes a work overload and the risk of reporting erroneous data. A CBHW report is 38-page long, so makes it a daunting task to complete and verify. Moreover, the head nurse does not always receive the offered DMT support for CBHW supervision and investigation of rumours, including deaths in the community.

Also, late payment of financial incentives may also influence CHBWs’ motivation to carry out in-depth investigations of rumours. According to several studies, lack of in-depth investigations may lead to infodemics, which refers to false or misleading information that may in turn cause widespread public reluctance to adopt the required infection control measures promoted by health authorities – thus delaying essential interventions [42–46].

Several guidelines exist for the completion of standardized forms for surveillance. However, they do not specify the means by which the reported data should be verified. This creates a risk of entering erroneously aggregated data that is inconsistent with the local reality. In turn this may lead to poor resource estimation, poor planning and performance assessment and, eventually, poor decision-making at central level. Innovating through the use of online forms or electronic applications could in this case, make it possible to better supervise the completion of forms at community level. That would promote timely detection of erroneous or incomplete data [47, 48].

In the specific case of diseases with an epidemic potential, the multi-skills requirement of ICPs, including routine activities, epidemiological surveillance, administration and management of resources, community collaboration, etc., generates a critical need for training and in-service training to ensure the timely identification and correct completion of surveillance forms. The lack of training of newly assigned ICPs by DMT members on surveillance procedures may be an additional explanation of the poor completion and filling level of surveillance forms.

Strategies such as on-site training should also be devised to engage all staff at health facilities, not just ICPs, in surveillance activities.

#### Processes categories

In a previous study in Burkina Faso, Geers and colleagues (2018) identified the cell phone as the main tool for CBHWs to report unusual events [24]. This corroborates our findings, where this tool was used in case of transport constraints or for immediate notification of unusual events. However, the cell phone report is usually followed by a hard copy (paper) transmission of epidemiological information between the PHC and health districts. Some actors pointed out this procedure as leading to an overload of work or a double activity. That calls for a reflection on the opportunities to improve data transmission and archiving of weekly and monthly reports including digital means.

We observed a lack of simplified case definition for some disease and skills, which could explain the misidentification of suspected cases or under-detection of cases by CBHWs and health facility workers. Hence the importance of training and the use of case definitions that are understandable at local level. Issues such as understanding of forms and/or case definitions could be addressed by using a local language during training sessions of community actors or by the use of translated forms, as recently applied in the case of community management of childhood infections and illnesses in the District of Barsalogho [49]. In Nigeria, Nnebue and colleagues (2012) recommended regular training and in-service training of relevant health care workers with adequate and regular provision of IDSR forms, copies of the standard case definitions, and other necessary logistics to the PHC [50]. These additional training activities by local and state governments imply the availability of financial resources, which may require considerations as to relevant reallocation opportunities.

We observed that factors such as the poor quality of the telephone network coverage or the unavailability of financial resources affect reporting. This situation sometimes lead actors across all levels to adopt accommodative methods, in order to report information in time or to promote active feedback to the community level. These accommodative methods included the use of private telephones and/or telephone units and use of any available means of transportation such as privately owned cars or motorcycles. These choices imply a cumbersome process of reimbursement. In their study from Zanzibar, Saleh and colleagues (2021) reported similar findings in terms of availability of communication services, inadequate transportation capacities and funds, which hindered regular supervision, training, and outbreak investigation [51].

The ministry of health is planning to deploy innovative monitoring tools such as RapidSMS^4^ and REC^5^ [52] for pilot diseases and should hopefully replace the current accommodative measures. However, for the new tools to be efficient, they must be appropriated by all actors in order to improve the overall performance of the system.

We also observed that all data analyses were undertaken at district or regional level, as PHC workers did not perform any analysis or interpretation of collected data at local level. As Rasmussen (2018) [53] pointed out, the inability of actors to analyse and work with data at the local level may negatively affect the quality of the data reported to the district level. In this study, we noted several forms with missing or inadequate data submitted to the District level which, given local analysis, may have been noted.

Additionally, epidemic management meetings could play a catalytic role for planning and decision-making at the local level. Yet, are increasingly difficult to maintain due to lack of financial means across all levels (regional, provincial, departmental, municipal). The current strategy of integrating these meetings with other activities, such as sexual and reproductive health and malaria control programmes, faces the challenge of a limited attendance numbers by all stakeholders. As a consequence key actors are excluded from essential discussions and decision-making that align with local expectations, such as financial support.

We identified a lack of direct laboratory feedback from the District to the PHCs in the case of a negative test result. This represents a motivational challenge, also noted by Drabo and colleagues (2015), who reported that without regular communication of test results, the relevance of notifying cases and the sense of useful contribution towards the system gets questioned at community level [54]. In fact, this feedback definitely encourages health workers and the community “rapporteurs” to participate in the surveillance system and in-service training [54].

#### Output categories

At the national level aggregated data are translated into yearbooks, statistical yearbooks, epidemiological reports and other reference documents that are distributed at district level. The dissemination of these documents may vary between districts. These resources, in reality, are used for service and system planning and management, advocacy and policy development at district level. However, nationally aggregated data may not adequately reflect the health situation in a given district, thus posing a challenge to the effectiveness of the developed policies and plans. Whereas the HMN [2] states that local information should be used to guide local decision-making, the capacity to analyse data often lacks at the peripheral levels where data are generated and where the results should be used for planning and management. That is sometimes, due to a poor archiving system and a lack of corrective actions in local databases after data validation sessions. In their analysis of the WHO-African region, Mbondji and colleagues (2014) underlined that health management information systems generate considerable data, but the information is rarely used because of concerns about bias, quality and timeliness [55].

## Conclusion

The reorganization of the health information system of Burkina Faso, with the inclusion of a stronger focus on emerging epidemics and CBHWs participation, would improve its performance in terms of epidemiological surveillance. In fact, even if the health system has guides and manuals for health information management, a well-defined information circuit and an internet platform for the storage of health data, we remarked that these are not enough. There is a need, to hold regular training / retraining sessions for agents involved in surveillance. To introduce a data quality control system, it is recommended to cultivate a habit of systematic search for missing data, among all surveillance actors, in order to continuously improve the quality of the epidemiological databases. It is also a good idea to ensure the development of simplified definitions of cases for emerging diseases, to include more local languages during training sessions of community actors. That could help those actors to reach a better understanding of signs, symptoms, indicators for a timely detection, notification of community case by CBHWs and ICPs. In addition, the system should also consider generating adapted means for CBHWs to carry out "autonomous" detection and rapid notification of cases in their communities. The encouragement of people involved at the peripheral level in routine activities of collecting and storing data should be extended to analysis and interpretation of data by local actors, with a view to further stimulate the use of local data. Health professionals, system managers or statisticians should not be the only users of health data. Indeed, those responsible for data collection should also benefit from its use.

## Data Availability

The data that support the findings of this study are available from Emerging EPIDEMICS project but restrictions apply to the availability of these data, which were used under license for the current study, and so are not publicly available. The datasets used and/or analysed during the current study are available from the corresponding author on reasonable request.

## Abbreviations

ABBEF: Burkinabe Association for Family Welfare
AIDS: Acquired Immunodeficiency Syndrome
CBHW: Community-based health worker
CBOA: Community-based organization animators
CISSE: Health information and epidemiological surveillance center/officer
CoGes: health facility management committee
CSD: District Health Council
CSPS: Primary health care center
CM/CMA: Medical center
DGESS: General directorate of studies and sectorial statistics
DMT: District management team
DPSP: Directorate for the Protection of Population Health
EnDoS: Health data warehouse
HD: Health district
HIS: Health information system
HMN: Health Metrics Network
ICP: Head nurse of PHC
IDSR: Integrated disease surveillance and response
IPC Burkina: Private and community initiative Burkina
MCD: District’s medical officer
NGO: Non-governmental organizations
PHC: Primary health care center
RMA: Monthly reports
SIREP: Epidemiological research and planning information service
SPIH: Hospital planning and information service
STELaB: System for Tracking Epidemiological Data and Laboratory Specimens.
TLOH: Telegram weekly official letter (Weekly reports)
WHO: World health organization

## Footnotes

1 The community based-organization (CBO) animator or monitoring and evaluation officer is employed by an NGO or community association, yet has an active role in the health system.

2 Integrated Disease surveillance and response

3 System for Tracking Epidemiological Data and Laboratory Specimens

4 women receiving mHealth-supported antenatal care in a village, from community based health workers

5 World Health Organization (WHO) Integrated Management of Childhood Illness (IMCI) method and strategy through computerization of medical consultation for children under five years

## References

[1] AbouZahr C, Boerma T. Health information systems: the foundations of public health. Bull World Health Organ. 2005;6. PMC2626318 16184276

[2] WHO, Health Metrics Network. Framework and standards for country health information systems. 2nd Edition. 2008.

[3] Ndongo JS, Ongolo-Zogo P. Policy brief: Renforcer le système d’information sanitaire pour accélérer la viabilisation du District de santé. 2010;

[4] WHO. Priority interventions to strengthen national health information systems. 2004 p. 16.

[5] WHO - Africa. Technical Guidelines for Integrated Disease Surveillance and Response in the WHO African Region, booklet one: Introduction section. 3rd Edition; 2019.

[6] Phalkey RK, Yamamoto S, Awate P, Marx M (2015). Challenges with the implementation of an Integrated Disease Surveillance and Response (IDSR) system: systematic review of the lessons learned. Health Policy Plan.

[7] Ministère de la santé, Burkina Faso. Guide technique pour la surveillance intégrée de la maladie et la riposte au Burkina Faso (Section 1 à 8: Etapes de la surveillance). 2012.

[8] Kebe MR, Ouangaré A, Tohouri R-R, Kouassi C, Barry MA, Chauffour J (2020). Evaluation de la performance du système d’information sanitaire de routine (PRISM) au Burkina Faso.

[9] Common Context Analysis (CCA) of Non-Governmental Cooperation Actors, Burkina Faso. 2015.

[10] Burkina Faso Health Profile - Burkina Faso Open Data [Internet]. [cité 9 févr 2021]. Disponible sur: https://burkinafaso.opendataforafrica.org/jyepxl/burkina-faso-health-profile. Accessed 9 Feb 2021.

[11] Ministère de la santé, Burkina Faso. Profil sanitaire complet du Burkina Faso. Module 2: Système de santé du Burkina Faso. 2017.

[12] Ministère de la santé, Burkina Faso. Plan national de développement sanitaire 2011 - 2020. 2011.

[13] Ministère de la santé, Burkina Faso. Annuaire statistique 2019. 2020.

[14] Ministère de la santé, Burkina Faso. Plan stratégique du système national d’information sanitaire 2010 - 2020. 2010.

[15] Ministère de la santé, Burkina Faso. Annuaire statistique 2018. 2019.

[16] Ministère de la santé, Burkina Faso. Description de postes des agents chargés du SNIS (1ère Edition). 2015.

[17] WHO. Integrated Disease Surveillance and Response in the African Region-a Guide for Establishing Community Based Surveillance. WHO Reg Off Afr. 2014.

[18] Ministère de la santé, Burkina Faso. Module de formation des agents de santé à base communautaire. 2016.

[19] Ministère de la santé, Burkina Faso (2017). Guide de remplissage du rapport mensuel d’activité de l’agent de santé à base communautaire.

[20] Ministère de la santé, Burkina Faso (2017). Guide de remplissage du rapport mensuel d’activités des CSPS/ Dispensaire/ Maternité/ CSI/ Clinique d’accouchement..

[21] Ministère de la santé, Burkina Faso (2001). Guide de remplissage des supports de collecte de données - Niveau CSPS, CM/CMA et ECD.

[22] Ministère de la santé, Burkina Faso, USAID, CDC, JHPIEGO (2018). Guide national de supervision des agents de santé à base communautaire (ASBC).

[23] Ministère de la santé, Burkina Faso (2016). Guide technique pour la surveillance intégrée de la maladie et la riposte au Burkina Faso. (Section 9: Directives relatives aux maladies, affections prioritaires et autres évènements de santé publique).

[24] Geers E, Sawadogo I, Nzietchueng S, Eugene Y (2018). Evaluation rapide des systèmes de surveillance des maladies à potentiel épidémique et épizootique dans une Région du Burkina Faso.

[25] Niamba L (2020). Geographical and Gender Disparities in the Registration of Births, Marriages, and Deaths in the Nouna Health and Demographic Surveillance System, Burkina Faso.

[26] District sanitaire de Dandé (2018). Plan d’action 2018 du District sanitaire de Dandé.

[27] District sanitaire de Dandé (2018). Plan d’action 2019 du District sanitaire de Dandé.

[28] Ndiaye SM, Quick L, Sanda O, Niandou S (2000). The value of community participation in disease surveillance: a case study from Niger. Health Promot Int..

[29] World Bank. Étude sur le Secteur Privé de la Santé au Burkina-Faso. 2012; Disponible sur: https://elibrary.worldbank.org/doi/abs/10.1596/978-0-8213-9701-5. Accessed 26 May 2021.

[30] Houéto D, Valentini H (2014). La promotion de la santé en Afrique : histoire et perspectives d’avenir. Santé Publique.

[31] Musso P. Critique des réseaux [Internet]. Presses Universitaires de France; 2003 [cité 3 sept 2020]. Disponible sur: https://www.cairn.info/critique-des-reseaux%2D%2D9782130501374. Accessed 3 Sept 2020.

[32] Grosjean S, Bonneville L (2007). Logiques d’implantation des TIC dans le secteur de la santé. Rev Fr Gest.

[33] Organisation Mondiale de la Santé, Bureau régional de l’Afrique. Guide technique pour la surveillance intégrée de la maladie et la riposte dans la région africaine de l’OMS: Volume 4 - Sections 8 et 9. 3ème Edition; 2019.

[34] Freifeld CC, Chunara R, Mekaru SR, Chan EH, Kass-Hout T, Iacucci AA (2010). Participatory Epidemiology: Use of Mobile Phones for Community-Based Health Reporting. PLOS Med.

[35] USAID, AIDSTAR-Two. L’utilisation des technologies de l’information et de la communication pour les programmes de planification familiale, santé de la reproduction et autres programmes de santé: une étude des tendances et données factuelles [Internet]. Disponible sur: https://www.msh.org/sites/msh.org/files/AIDSTAR-Two_ICT_FP_Paper_-French_11-15-11. Accessed 9 Feb 2021.

[36] Nsaghurwe A, Dwivedi V, Ndesanjo W, Bamsi H, Busiga M, Nyella E, et al. One country’s journey to interoperability: Tanzania’s experience developing and implementing a national health information exchange. BMC Med Inform Decis Mak [Internet]. 2021 [cité 15 juin 2021];21. Disponible sur: https://www.ncbi.nlm.nih.gov/pmc/articles/PMC8086308. Accessed 15 June 2021. 10.1186/s12911-021-01499-6 PMC8086308 33926428

[37] Odhiambo-Otieno GW (2005). Evaluation of existing District Health Management Information Systems : A case study of the District Health Systems in Kenya. Int J Med Inf.

[38] Ouedraogo B. Système de surveillance épidémiologique au Burkina Faso : Contribution à la mise en place d’un dispositif informatisé de remontée des données du paludisme et analyses géo-épidémiologiques pour la prise de décision [Thèse]. Université D’Aix-Marseille; 2018.

[39] Lefaso.Net. Secteur de la Santé au Burkina : 2019, une année mouvementée ! [Internet]. [cité 12 mai 2021]. Disponible sur: https://lefaso.net/spip.php?article94144. Accessed 12 May 2021.

[40] Schweyer F-X (2005). Working in Network: An Ambiguous Consensus and a Lack of Tools. Sociol Prat..

[41] Cabe M-H. La santé en réseaux. Quelles innovations ? Santé En Réseaux Quelles Innov. 2005;(11).

[42] WHO (2018). Managing epidemics: key facts about major deadly diseases.

[43] Sell TK, Hosangadi D, Trotochaud M (2020). Misinformation and the US Ebola communication crisis: analyzing the veracity and content of social media messages related to a fear-inducing infectious disease outbreak. BMC Public Health.

[44] Kutalek R, Baingana F, Sevalie S, Broutet N, Thorson A (2020). Perceptions on the collection of body fluids for research on persistence of Ebola virus: A qualitative study. PLoS Negl Trop Dis..

[45] Sumo J, George G, Weah V, Skrip L, Rude JM, Clement P (2019). Risk communication during disease outbreak response in post-Ebola Liberia: experiences in Sinoe and Grand Kru counties. Pan Afr Med J..

[46] Fung IC-H, Fu K-W, Chan C-H, Chan BSB, Cheung C-N, Abraham T, et al. Social Media’s Initial Reaction to Information and Misinformation on Ebola, August 2014: Facts and Rumors. Public Health Rep Wash DC 1974. juin 2016;131(3):461-73. 10.1177/003335491613100312 PMC4869079 27252566

[47] Organisation Mondiale de la Santé, Bureau régional de l’Afrique. Guide technique pour la Surveillance Intégrée de la maladie et la riposte dans la région africaine: Volume 4 - Sections 8 et 9. 3ème Edition; 2019.

[48] Ahmed K, Bukhari MAS, Dauod MA, Lugala PC, Popal GR, Abouzeid A, et al. Development and Implementation of Electronic Disease Early Warning Systems for Optimal Disease Surveillance and Response during Humanitarian Crisis and Ebola Outbreak in Yemen, Somalia, Liberia and Pakistan. Online J Public Health Inform. 2019;11(2):e11. 10.5210/ojphi.v11i2.10157 PMC6788902 31632605

[49] Ministère de la santé, Burkina Faso, Seck A, Valéa D. Analyse de la santé communautaire au Burkina Faso [Internet]. 2011 févr. Disponible sur: https://www.unicef.org/bfa/french/analyse_de_la_sante_communautaire_au_Burkina_Faso. Accessed 8 Feb 2021.

[50] Nnebue CC, Onwasigwe CN, Adogu POU, Onyeonoro UU (2012). Awareness and knowledge of disease surveillance and notification by health-care workers and availability of facility records in Anambra state, Nigeria. Niger Med J J Niger Med Assoc..

[51] Saleh F, Kitau J, Konradsen F, Mboera LEG, Schiøler KL (2021). Assessment of the core and support functions of the integrated disease surveillance and response system in Zanzibar, Tanzania. BMC Public Health.

[52] TIC et Santé : Concertation entre acteurs pour améliorer la qualité des services de santé par les technologies de l’information et de la communication - leFaso.net, l’actualité au Burkina Faso [Internet]. [cité 30 juill 2020]. Disponible sur: https://lefaso.net/spip.php?article70123. Accessed 30 July 2020.

[53] Rasmussen SL (2018). Plans and “off-plan activities”: Exploring the roles of data and situated action in health planning in Burkina Faso. E J Info Sys Dev Ctries [Internet].

[54] Poliomyelitis Case Surveillance Data Management in Burkina Faso | Cairn International Edition [Internet]. [cité 7 sept 2020]. Disponible sur: https://www.cairn-int.info/journal-sante-publique-2015-6-page-855. Accessed 7 Sept 2020.

[55] Mbondji PE, Kebede D, Soumbey-Alley EW, Zielinski C, Kouvividila W, Lusamba-Dikassa P-S (2014). Health information systems in Africa: descriptive analysis of data sources, information products and health statistics. J R Soc Med.

